# Reality shock and its associated factors among newly graduated nurses in China: A cross-sectional study

**DOI:** 10.1371/journal.pone.0350041

**Published:** 2026-05-26

**Authors:** Liping Li, Zhangyi Wang, Xiaochun Tang, Jun Qu, Huifang Zhou, Lamei Chen, Hongxia Wu, Shuguang Tan, Mengsu Liu

**Affiliations:** 1 Nursing Department, Gastrointestinal Surgery/ National Key Clinical Construction Specialist General Surgery, The Affiliated Hengyang Hospital of Hunan Normal University & Hengyang Central Hospital, Hengyang, Hunan, China; 2 Department of Respiratory and Critical Care Medicine, Shanxi Provincial People’s Hospital, Taiyuan, Shanxi, China; University of Basel Institute for Biomedical Ethics: Universitat Basel Institut fur Bio- und Medizinethik, SWITZERLAND

## Abstract

**Background:**

Newly graduated nurses often experience a transition period marked by reality shock, which can adversely affect their professional retention and career development. However, the associated factors remain incompletely understood, and there is a notable gap in evidence regarding the role of the impostor phenomenon in this context.

**Objectives:**

To investigate the reality shock among newly graduated nurses and identify its associated factors, with a specific focus on the impostor phenomenon.

**Methods:**

A cross-sectional design was implemented, and the high-quality reporting of the study adhered to the Strengthening the Reporting of Observational Studies in Epidemiology (STROBE) Statement. From July 2024 to January 2025, a total of 381 newly graduated nurses were recruited from 3 tertiary grade-A hospitals in China by convenience sampling. The inclusion criteria required nurses to hold a valid nurse qualification certificate, be registered at the hospital, have a clinical working time of ＜ 1 year, and provide informed consent while cooperating with investigators. Exclusion criteria applied to hospital trainees, standardized training personnel, and personnel not on duty for various reasons. The Demographic Characteristics Questionnaire, Reality Shock Scale for Newly Graduated Nurses, and Clance Impostor Phenomenon Scale were used. The data was analyzed using descriptive statistics, univariate analyses, correlation, and multiple linear regression analysis.

**Results:**

The total scores for reality shock and impostor phenomenon were 93.66 ± 38.94 and 44.19 ± 16.94, respectively. Reality shock was positively correlated with the impostor phenomenon (*r* = 0.635, *p* < 0.01). Multiple linear regression analysis showed that educational level, monthly income, number of night shifts per month, and impostor phenomenon were the main factors affecting the reality shock among newly graduated nurses (all *p* < 0.01), accounting for 51.80% of the total variance.

**Conclusion:**

This study identified a moderately high level of reality shock among newly graduated nurses. Key determinants associated with this shock included educational level, monthly income, number of night shifts per month, and impostor phenomenon. Nursing management should develop targeted interventions addressing these modifiable factors, particularly impostor phenomenon, which may help mitigate reality shock and facilitate successful career adaptation among newly graduated nurses.

## 1. Introduction

Newly graduated nurses, defined as registered nurses within their first year of clinical practice post-licensure, represent a vital cohort and emerging force within the global healthcare workforce [1]. This group undergoes a critical transition from academic preparation to professional practice, necessitating rapid adaptation to complex clinical environments, assimilation of workplace cultures, and acquisition of advanced competencies to meet dynamic patient care demands [2]. Empirical investigations [3,4] consistently demonstrate that a substantial proportion of newly graduated nurses experience significant reality shock during their inaugural year of employment. This phenomenon arises from a pronounced dissonance between pre-employment expectations and the exigencies of actual clinical responsibilities. As operationally defined in the literature, reality shock refers to a constellation of adverse sociocultural, physical, and emotional reactions triggered by immersion in unfamiliar clinical environments. Furthermore, in the context of newly graduated nurses, it denotes a perceived misalignment between their preformed expectations and the actual realities of clinical practice—that is, a dissonance arising when their on-the-ground experiences do not match what they had anticipated. Critically, cumulative evidence [5–7] indicates that unmitigated reality shock frequently culminates in workplace maladjustment, manifesting as heightened anxiety, pervasive negativity, perceived helplessness, professional frustration, and impaired role transition.

Confronted with the high-intensity demands of frontline clinical work, newly graduated nurses often encounter deficits in theoretical application, procedural skill proficiency, clinical experience, and communication efficacy [3,8]. This gap frequently results in a pronounced imbalance between their psychological expectations and the realities of practice, thereby precipitating reality shock. Furthermore, the dissonance between their initial clinical experiences and longer-term career aspirations can exacerbate this shock, aggravate self-ability denial [9]. High levels of reality shock are strongly associated with negative psychological sequelae, including anxiety and depression, significantly elevating the risk of attrition. Supporting this, empirical studies reveal that junior nurse turnover rates substantially exceed those of experienced nurses [10,11], with reports indicating that approximately 20.1% to 56.1% of newly graduated nurses express strong turnover intentions [12]. This instability critically undermines the continuity of hospital nursing teams and ultimately jeopardizes patient safety and treatment outcomes. Additionally, Labrague et al. [13] demonstrated that newly graduated nurses’ reality shock not only diminished their capacity for humanistic care but also correlated with a higher incidence of nursing practice errors (e.g., interruptions). Such outcomes often provoke critical reassessment of career choices, subsequently fostering intentions to transfer units or exit the profession entirely. These sequelae not only compromise patient care quality but also exacerbate nursing shortages and inflate institutional training and recruitment expenditures, may leading to the impostor phenomenon [14].

The impostor phenomenon describes a psychological pattern in which individuals persistently attribute their accomplishments to external factors—such as luck, interpersonal appeal, or assistance from others—rather than to their own capabilities or efforts [15]. Psychologists conceptualize impostor phenomenon as a genuine manifestation of self-doubt [16], increasingly recognized as a subclinical cognitive trait [17]. Research [18,19] indicates that impostor phenomenon is widespread, with notably high prevalence observed among newly graduated nurses [15]. This phenomenon exerts multifaceted detrimental effects on nurses: it fosters reluctance to voice opinions or initiate change in clinical settings and undermines accurate self-assessment, thereby constraining professional development and the realization of their full potential. Furthermore, impostor phenomenon triggers maladaptive behaviors such as excessive preparation, task procrastination, and heightened fear of failure. These responses contribute to chronic anxiety, accelerated job burnout, and diminished work efficiency, ultimately compromising both psychological well-being and occupational performance, which may be a predictor of reality shock among newly graduated nurses [19].

In summary, the development of evidence-based interventions to effectively mitigate reality shock among newly graduated nurses, enhance their professional identity, reduce the impostor phenomenon, and ultimately improve patient care quality represents a critical priority for both research and clinical practice, warranting significant attention [20]. Healthcare administrators must proactively identify the systemic and individual challenges inherent in this vulnerable transition period and establish comprehensive, multifaceted support systems. Such initiatives are crucial to safeguarding both high standards of patient care and ensuring workforce stability.

However, empirical research investigating the specific relationship between reality shock and impostor phenomenon among newly graduated nurses remains notably limited. Furthermore, there has been scant empirical investigation into the experience of reality shock among newly graduated nurses within the specific context of the Chinese healthcare system. Therefore, this study aims to investigate reality shock among Chinese newly graduated nurses, analyze the impact of demographic factors and impostor phenomenon on reality shock, and to reduce reality shock and impostor phenomenon of newly graduated nurses, thereby facilitating the development of targeted interventions to mitigate these challenges.

To address these gaps, the present study introduces several key innovations. First, it represents one of the first empirical attempts to quantitatively examine the specific relationship between reality shock and the impostor phenomenon within the unique and under-researched context of the Chinese healthcare system. Second, by identifying the demographic factors that influence reality shock and analyzing the role of the impostor phenomenon as a potential predictor, this research provides a more nuanced understanding of the mechanisms underlying reality shock. Ultimately, the findings are expected to offer a novel, evidence-based foundation for developing culturally sensitive and precisely targeted interventions aimed at mitigating reality shock, alleviating the impostor phenomenon, and supporting the successful transition and retention of newly graduated nurses in China.

## 2. Objective

This study aims to investigate the reality shock among newly graduated nurses and identify its associated factors, with a specific focus on the impostor phenomenon.

## 3. Methods

### 3.1 Study design and setting

This study employed a cross-sectional design to examine the prevalence and associations between the impostor phenomenon, reality shock, and other relevant variables among newly graduated nurses in China at a single point in time. The study reporting adhered to the Strengthening the Reporting of Observational Studies in Epidemiology (STROBE) guidelines for cross-sectional studies.

### 3.2 Participants and sample

A convenience sampling method was utilized to recruit participants from three tertiary grade-A hospitals in Hunan Province, China, between July 2024 and January 2025. This approach was adopted primarily due to its feasibility and efficiency in accessing the target population within the practical constraints of the study. The inclusion criteria stipulated that participants must: (1) hold a valid nurse qualification certificate, (2) be officially registered nurses at the involved hospitals, (3) have less than one year of clinical working experience, and (4) provide informed consent. Exclusion criteria encompassed hospital trainees, nurses undergoing standardized training programs, and those absent from duty for various reasons during the data collection period. It is acknowledged that this non-probability sampling method may limit the sample’s representativeness of the broader population of newly graduated nurses in China and could introduce selection bias. Consequently, the identified factors and associations should be interpreted as specific to the context and characteristics of this particular sample.

The minimum sample size required for this study was determined using F-tests in G*Power software (version 3.1.9.7) [21], with the α error probability set at 0.05 and statistical power (1 – β) at 95%. As all variables in the study are continuous, the analysis was conducted using the “Linear multiple regression: Fixed model, R² deviation from zero” test option. An a priori power analysis was selected to compute the required sample size given α, power, and effect size. With an effect size f² of 0.15, α = 0.05, and 1 – β = 0.95, the calculated minimum sample size was 107.

Considering the practical constraints of the hospital and clinical setting, a total of 381 participants were ultimately enrolled. A post hoc power analysis was then performed using the same statistical test and an effect size f² of 0.15, α = 0.05, and the actual sample size of 381. The computed power (1 – β) reached 1.00, exceeding the initially set value of 0.95, which indicates that the enrolled sample size was sufficient for the planned mediation analysis.

### 3.3 Measurements

#### 3.3.1 The Demographic Characteristics Questionnaire.

A self-developed Demographic Characteristics Questionnaire was utilized to collect data on seven demographic variables, including age, gender, educational level, marital status, monthly income, number of night shifts per month, and whether is a only child. This questionnaire was developed based on a comprehensive review of the relevant literature.

#### 3.3.2 The Reality Shock Scale for Newly Graduated Nurses (RSSNGNs).

The scale was originally developed by Çiriş Yildiz et al. [22]. Subsequently, Wu et al. [23] performed the translation and cross-cultural adaptation to create the Chinese version. The instrument comprises 41 items distributed across four distinct dimensions: job performance (3 items), competency (6 items), professional knowledge (11 items), and relationships and cooperation (21 items). Responses are captured using a 5-point Likert scale, ranging from 1 (“never”) to 5 (“always”). The total score ranges from 41 to 205, with higher scores reflecting a greater reality shock. The scale demonstrated high internal consistency, with a Cronbach’s α of 0.960 in Wu et al.’s [23] study and 0.951 in the current study.

#### 3.3.3 The Clance Impostor Phenomenon Scale (CIPS).

The scale was originally developed by Clance [24]. A Chinese adaptation of this scale was subsequently validated by Jiang et al. [15]. This self-report instrument comprises 18 items distributed across three dimensions: self-doubt (8 items), external attribution (6 items), and passive camouflage (4 items). Responses were recorded using a 5-point Likert scale ranging from 1 (“strongly disagree”) to 5 (“strongly agree”). Total scores range from 18 to 90, with higher scores indicating greater imposter phenomenon. The scale demonstrated high internal consistency, with a Cronbach’s α of 0.930 in Jiang et al.’s [15] study and 0.935 in the current study.

### 3.4 Data collection

The data collection process consisted of a pilot study followed by the main investigation. A pilot study was first conducted with a convenience sample of 30 newly graduated nurses (who were excluded from the main study) to assess feasibility, clarity of instructions, item comprehensibility, appropriateness of response formats, estimated completion time, and overall data collection logistics. Based on pilot feedback, minor revisions to the wording of two items were made to improve clarity. No significant procedural issues were identified.

For the formal investigation, approval was obtained from the participating hospitals. With the assistance of the head nurse in each department, potential participants were approached. Researchers provided unified instructions, explaining the study’s purpose, significance, and the confidentiality of their responses. Participants then completed the questionnaire package face-to-face via an online platform. To ensure data quality, several control measures were implemented: (1) system settings restricted each participant to a single submission; (2) all questions were mandatory to prevent missing data; (3) questionnaires completed in less than one minute were deemed invalid and excluded; (4) questionnaires with obviously contradictory or inconsistent responses (e.g., logically conflicting answers or mismatched personal information) were also excluded during the data cleaning process. These rigorous quality control procedures resulted in the distribution of 400 questionnaires and the collection of 381 valid responses, yielding an effective response rate of 95.4%.

### 3.5 Statistical analysis

Two researchers independently recorded and systematically analyzed the raw data using EpiData (version 3.1) and IBM SPSS Statistics (version 25.0). Descriptive statistics, presented as frequencies (n) and percentages (%), characterized the demographic profiles of participants. The normality of continuous variables was assessed using the Shapiro-Wilk test. Normally distributed continuous variables were expressed as mean ± standard deviation (Mean ± SD). Inter-group comparisons for normally distributed data utilized independent samples t-tests or one-way analysis of variance (ANOVA), as appropriate. For continuous variables violating normality, data were summarized as median and interquartile range (IQR), with group comparisons performed using the Mann-Whitney U test (two groups) or the Kruskal-Wallis H test (≥3 groups). To identify factors influencing reality shock among newly graduated nurses, Pearson correlation analysis was conducted first, followed by multiple linear regression analysis. Given that the dependent variable (reality shock total score) is derived from Likert-scale items and may not strictly meet the assumption of continuous measurement, we also performed a sensitivity analysis using a Generalized Linear Model (GLM) with a gamma distribution and log link function to confirm the robustness of our findings. Multicollinearity was assessed using variance inflation factors (VIF), with a VIF < 5 indicating no significant multicollinearity. Additionally, potential interactions between significant predictors (educational level, monthly income, night shifts, and impostor phenomenon) were tested by including interaction terms in the regression model. No significant interactions were found (all *p* > 0.05). Statistical significance was defined as *p* < 0.05 (two-tailed) for all analyses.

### 3.6 Ethics approval and consent to participate

The study was approved by the Ethics Committee of Hengyang Central Hospital (Approval No: HYZXYY-2024–59). This study was conducted in accordance with the principles of the Declaration of Helsinki and adhered to relevant guidelines and regulations. First, potential participants were identified and approached by the research team with the assistance of the chief nurse, following authorization from hospital administrators. Second, prior to data collection, researchers provided participants with comprehensive information regarding the study’s aim, significance, assurance of confidentiality, and data handling procedures. Participants were informed that their participation was voluntary and that they retained the right to withdraw at any time without consequence. Additionally, they were assured that all collected information would be used solely for academic research purposes and not for commercial gain. Written informed consent was subsequently obtained from all participants before the commencement of the study.

## 4. Results

### 4.1 Demographic characteristics and univariate analysis of reality shock

A total of 381 newly graduated nurses were recruited. The mean age of the participants was 23.25 years (SD = 1.19). In terms of age distribution, 65 (17.06%) were ≤22 years old, 298 (78.22%) were 23 ~ 25 years old, and 18 (4.72%) were ＞25 years old. The gender distribution was as follows: 29 (7.61%) were male, and 352 (92.39%) were female. Other demographic characteristics and the results of the univariate analysis are presented in Table 1.

**Table 1:** Demographic characteristics and univariate analysis of reality shock among newly graduated nurses (*n* = 381, M ± SD).

Characteristics	*n*	%	Total score of RSSNGNs	*t*/ *F*	*p*
**Age (years old)**				1.118	0.206
≤ 22	65	17.06	97.21 ± 31.63		
23 ~ 25	298	78.22	94.18 ± 36.52		
> 25	18	4.72	88.75 ± 30.97		
**Gender**				0.420	0.674
Male	29	7.61	96.59 ± 35.11		
Female	352	92.39	93.42 ± 39.28		
**Educational level**				4.765	0.003
Technical secondary	2	0.52	126.50 ± 34.95		
Junior college	163	42.78	86.38 ± 32.02		
Undergraduate	202	53.02	97.66 ± 43.02		
Master degree or above	14	3.68	116.07 ± 36.31		
**Marital status**				0.402	0.688
Married	41	10.76	95.98 ± 41.06		
Unmarried	340	89.24	93.38 ± 38.74		
**Monthly income (RMB)**				5.591	0.001
< 2000	185	48.56	96.20 ± 36.57		
2000 ~ 3999	51	13.39	107.71 ± 42.53		
4000 ~ 6000	113	29.65	82.83 ± 39.32		
> 6000	32	8.40	94.84 ± 36.87		
**Number of night shifts per month**				4.536	0.001
No	144	37.80	87.98 ± 35.59		
≤ 5	100	26.25	91.60 ± 35.07		
6 ~ 10	119	31.23	92.18 ± 42.32		
11 ~ 15	10	2.62	125.30 ± 46.33		
> 15	8	2.10	129.13 ± 8.05		
**Whether is a only child**				0.976	0.329
Yes	77	20.21	97.53 ± 39.39		
No	304	79.79	92.68 ± 38.83		

Note: RSSNGNs = The Reality Shock Scale for Newly Graduated Nurses.

The univariate analysis was conducted to identify factors associated with reality shock. As presented in Table 1, the results suggested that educational level, monthly income, and number of night shifts per month were significantly associated with reality shock among newly graduated nurses (all *p* < 0.01). It should be noted that the interpretation for the ‘technical secondary’ education subgroup (*n* = 2) requires extreme caution due to its very small sample size.

### 4.2 Scores of reality shock and impostor phenomenon among newly graduated nurses

The total score for reality shock was 93.66 ± 38.94, and the average reality shock score was 2.28 ± 0.95. Among four dimensions of the RSSNGNs, “Job performance” had the highest average score (2.50 ± 1.03), while “Professional knowledge” had the lowest (2.25 ± 0.96). The average scores of “Competency” and “Relationship and cooperation” were 2.31 ± 1.06 and 2.27 ± 0.99, respectively.

The total score for impostor phenomenon was 44.19 ± 16.94, and the average impostor phenomenon score was 2.54 ± 0.94. The average scores of the three dimensions of CIPS were “Self doubt” (2.52 ± 0.98), “Passive camouflage” (2.51 ± 1.02), and “External attribution” (2.34 ± 0.95) from high to low, respectively.

The RSSNGNs and CIPS scores were presented in Table 2.

**Table 2:** The scores of RSSNGNs and CIPS among newly graduated nurses (*n* = 381, M ± SD).

Dimensions	Number of items	Number of items	Average score of items	Ranking
**RSSNGNs total score**	41	93.66 ± 38.94	2.28 ± 0.95	—
Job performance	3	7.50 ± 3.08	2.50 ± 1.03	1
Competency	6	13.88 ± 6.33	2.31 ± 1.06	2
Professional knowledge	11	24.71 ± 10.61	2.25 ± 0.96	4
Relationship and cooperation	21	47.57 ± 20.74	2.27 ± 0.99	3
**CIPS total score**	18	44.19 ± 16.94	2.54 ± 0.94	—
Self doubt	8	20.13 ± 7.86	2.52 ± 0.98	1
External attribution	6	14.02 ± 5.72	2.34 ± 0.95	3
Passive camouflage	4	10.03 ± 4.09	2.51 ± 1.02	2

Note: RSSNGNs = The Reality Shock Scale for Newly Graduated Nurses; CIPS = The Clance Impostor Phenomenon Scale.

### 4.3 Correlation between reality shock and impostor phenomenon among newly graduated nurses

The total reality shock score was positively correlated with the total impostor phenomenon score (*r* = 0.635, *p* < 0.01), with positive correlations observed across all dimensions (*r* = 0.512–0.603, *p* < 0.01), as shown in Table 3.

**Table 3:** The correlation between reality shock and impostor phenomenon among newly graduated nurses (*n* = 381, *r*).

Item	CIPS total score	Self doubt	External attribution	Passive camouflage
**RSSNGNs total score**	0.635**	0.611**	0.610**	0.566**
**Job performance**	0.574**	0.569**	0.512**	0.532**
**Competency**	0.580**	0.550**	0.552**	0.537**
**Professional knowledge**	0.631**	0.603**	0.623**	0.564**
**Relationship and cooperation**	0.589**	0.570**	0.566**	0.516**

Note: **p ＜ 0.01; RSSNGNs = The Reality Shock Scale for Newly Graduated Nurses; CIPS = The Clance Impostor Phenomenon Scale.

### 4.4 Multiple linear regression analysis of reality shock among newly graduated nurses

Taking the reality shock total score among newly graduated nurses as the dependent variable, and the statistically significant general demographic characteristics and impostor phenomenon total score identified in the univariate analysis as independent variables (assignment details are presented in Table 4, a multiple linear regression analysis was performed. The results revealed that the significant predictors of reality shock among newly graduated nurses were educational level, monthly income, number of night shifts per month, and impostor phenomenon (*R* = 0.723, *R*² = 0.523, adjusted *R*² = 0.518, *F* = 165.31, *p* < 0.001). And the 95% confidence interval (CI) excluding 0, indicates that the difference was statistically significant (*p* < 0.001). Collectively, these predictors explained 51.80% of the variance in reality shock scores, as detailed in Table 5. In additional, a sensitivity analysis using a Generalized Linear Model (GLM) with a gamma distribution and log link function yielded similar results, confirming the robustness of the multiple linear regression findings. Additionally, no significant interactions were observed among the predictors (educational level, monthly income, night shifts, and impostor phenomenon).

**Table 4:** Independent variable assignment.

Project	Assignment method
Age	≤ 22 = 1, 22 ~ 25 = 2, ＞ 25 = 3
Gender	Male = 0, Female = 1
Educational level	Technical secondary = 1, Junior college = 2, Undergraduate = 3, Master degree or above = 4
Marital status	Married = 0, Unmarried = 1
Monthly income	＜2000 = 1, 2000 ~ 3999 = 2, 4000 ~ 6000 = 3, ＞ 6000 = 4
Number of night shifts per month	None = 1, ≤ 5 = 2, 6 ~ 10 = 3, 11 ~ 15 = 4, ＞ 15 = 5
Whether is a only child	Yes = 0, No = 1
CIPS total score	Original value entry

Note: CIPS = The Clance Impostor Phenomenon Scale.

**Table 5:** Multiple linear regression analysis of reality shock among newly graduated nurses (*n* = 381).

Project	*B*	*SE*	*β*	*t*	*p*	*95% CI*
Constant quantity	−3.213	5.525	—	−0.582	0.561	
Educational level	6.100	2.220	0.089	2.748	0.006	[1.75, 10.45]
Monthly income	−3.918	1.243	−0.107	−3.152	0.002	[-6.35, -1.48]
Number of night shifts per month	3.298	1.238	0.084	2.664	0.008	[0.87, 5.72]
CIPS total score	1.856	0.065	0.807	28.375	＜0.001	[1.73, 1.98]

Note: CIPS = The Clance Impostor Phenomenon Scale; *R* = 0.723, *R*² = 0.523, adjusted *R*² = 0.518, *F* = 165.31, *p* ＜ 0.001.

## 5. Discussion

### 5.1 The status quo of reality shock and impostor phenomenon among newly graduated nurses

This study revealed significantly elevated levels of reality shock and impostor phenomenon among newly graduated nurses in the investigated cohort. First, our observed levels of reality shock substantially exceeded those documented by Labrague et al. [20] among newly recruited ICU nurses and also surpassed the broader findings from Çiriş Yildiz et al. [22]. This pronounced disparity underscores the potential influence of contextual and systemic factors specific to the Chinese healthcare environment. Several interconnected factors warrant deeper consideration. Firstly, deeply ingrained societal perceptions within China, where nursing is often reductively viewed through the lens of basic tasks, likely contribute to diminished professional identity and exacerbate the dissonance between expectations and workplace realities for new graduates [25,26]. Secondly, the pervasive challenge of clinical nursing shortages in China creates a demanding work environment characterized by excessive workloads [27]. This chronic understaffing directly intensifies job strain and frustration, amplifying the experience of reality shock, a phenomenon consistent with Kramer et al.‘s [28] findings. Consequently, the transition experience for new nurses appears particularly vulnerable within this confluence of societal undervaluation, systemic resource constraints, and the intrinsic pressures manifesting as high job performance anxiety and impostor feelings.

Furthermore, a detailed analysis of the subscale scores provides deeper insights into the specific nature of these experiences. Regarding reality shock, the dimension of “Job performance” recorded the highest average score. This finding indicates that the primary domain of reality shock for these new nurses revolves around concerns and challenges related to their clinical competence and ability to perform their duties effectively. The transition from the theoretical knowledge and simulated environments of education to the unpredictable, high-stakes demands of actual patient care appears to be a central catalyst for their distress. Similarly, the profile of impostor phenomenon scores is highly revealing. The elevated scores in the “Self-doubt” and “Passive camouflage” dimensions are particularly noteworthy, as they reflect the core psychological and behavioral manifestations of this phenomenon. A high level of “Self-doubt” signifies these nurses’ persistent internal feelings of intellectual and professional fraudulence, despite their qualifications and accomplishments. Concurrently, the high score in “Passive camouflage” suggests a tendency to avoid evaluation and downplay their abilities, often by attributing their successes to external factors like luck, which is a classic behavioral pattern associated with the impostor phenomenon. The interpretation of these specific subscale scores strengthens the theoretical significance of our results, directly linking our empirical findings to the conceptual frameworks of reality shock and the impostor phenomenon.

To mitigate these challenges, interventions must extend beyond individual coping strategies. While supporting new graduates is important, systemic reforms are crucial [29,30]. In light of our findings, we propose a multi-pronged approach targeting both educational preparation and workplace integration to effectively mitigate RS and IP.

First, we strongly advocate for an enhanced emphasis on soft skills training within undergraduate nursing education. The integration of competencies such as self-efficacy, emotional regulation, critical reflection, resilience, and effective communication into the core curriculum is paramount. By proactively building these psychological resources pre-licensure, nursing programs can equip graduates with robust coping mechanisms, serving as a critical preventative measure against the onset of the impostor phenomenon and facilitating a smoother transition into professional practice.

Second, at the organizational level, we recommend that nursing management improve the clarity and realism of job profiles for newly graduated nurses (NGNs). A pronounced dissonance between pre-employment expectations and actual clinical responsibilities is a well-documented root cause of RS [28]. Therefore, job descriptions and competency profiles must transparently and accurately reflect the realities of the clinical environment. This clarity will help align NGNs’ expectations with their actual roles, thereby reducing the initial shock and frustration. Furthermore, we suggest a strategic shift towards competency-based human capital selection in the hiring process. Moving beyond traditional criteria, evaluating candidates against well-defined, job-specific competencies and behavioral requirements can ensure a better match between the new graduate’s skill set and the demands of the clinical setting. This strategy not only supports better organizational fit but also reduces the initial performance anxiety and stress that significantly contribute to RS.

Ultimately, addressing the core issues requires also requires concerted efforts to elevate the societal status of the nursing profession, alongside substantial investments in expanding the nursing workforce and implementing evidence-based workload management strategies. Only through such multi-level approaches can the profound reality shock and associated impostor phenomenon experienced by newly graduated nurses in this context be effectively alleviated.

### 5.2 Associated factors of reality shock among newly graduated nurses

#### 5.2.1 Educational level.

This study identified educational level as a significant predictor of reality shock among newly graduated nurses, a finding warranting deeper exploration. Although the sample size was exceedingly small (*n* = 2) and thus the result must be interpreted with utmost caution, the data indicated that newly graduated nurses with a technical secondary education might exhibit higher reality shock levels. This observation, albeit preliminary, appears to align conceptually with the observations of Yang et al. [31]. The pronounced impact, if replicable in larger samples, could stem from a critical misalignment: their foundational knowledge structure, skill proficiency, and cognitive preparation may be less robust relative to the demanding knowledge and skill requirements encountered in complex clinical environments. This disparity creates a significant competency gap, exacerbating feelings of mismatch and maladaptation. Interestingly, beyond the technical secondary level, our data suggest a positive association between higher educational level and elevated reality shock. This counterintuitive pattern may reflect heightened career expectations among more highly educated newly graduated nurses [32]. Their aspirations for professional growth and autonomy may be disproportionately greater than the often task-heavy, hierarchical realities of initial clinical roles, leading to disappointment and shock. This finding resonates with the work of Li et al. [33], who documented weaker professional self-concept among highly educated nurses, subsequently correlating with lower job satisfaction and increased turnover intention–adverse outcomes potentially amplified by the experience of reality shock [24].

Consequently, our findings underscore the critical need for differentiated support strategies tailored to the distinct vulnerabilities associated with different educational backgrounds. For technical secondary graduates, interventions must prioritize bridging the competency gap through intensive, structured skill development and knowledge reinforcement programs. For bachelor’s and higher degree holders, support should focus on realistic role orientation, expectation management, and fostering professional identity development to mitigate the disillusionment arising from unmet aspirations [34]. Evidence from Liu et al. [35], demonstrating the efficacy of specialized theoretical training, psychological counseling, and peer support in reducing reality shock among newly graduated nurses in specialized settings like stomatological hospitals, provides a valuable framework. Nursing managers should leverage such evidence-based approaches, designing onboarding and ongoing development programs that explicitly address the specific educational backgrounds, knowledge profiles, psychological predispositions, and work expectations of newly graduated nurses. Clarifying achievable career pathways within the clinical setting is also paramount to reducing RS across all educational strata.

#### 5.2.2 Monthly income.

This study revealed a non-linear relationship between monthly income and reality shock among newly graduated nurses. Specifically, nurses earning between 2000 and 3999 yuan experienced the highest levels of reality shock, while those within the 4000–6000 yuan bracket reported the lowest levels. This finding contrasts with Fang Ling’s [36] research on nurse career compromise, which suggested a more direct inverse relationship between income and negative work experiences. The discrepancy may stem from the highly variable compensation structures for newly graduated nurses across different hospitals in our setting, where base salaries and performance coefficients are institution-specific. Furthermore, the demographic profile of our cohort – predominantly unmarried, post-2000 generation individuals – likely moderated the perceived impact of income. Factors such as personal autonomy, single-child status, and favorable family backgrounds may have reduced immediate economic pressures and, consequently, the salience of income as a primary stressor related to reality shock. This interpretation aligns with Daehlen et al.’s [37] observation that, within the critical first three post-graduation years, nurses place comparatively less emphasis on high income and job security than other professionals like doctors and teachers, prioritizing other aspects of early career adaptation. Therefore, we recommend that nursing managers develop nuanced performance-based compensation strategies tailored to individual career development stages and adaptability. Concurrently, targeted support and guidance should be offered based on the varying levels of economic pressure faced by newly graduated nurses to more effectively mitigate reality shock.

#### 5.2.3 Number of night shifts per month.

This study demonstrates a significant association between increased number of night shifts per month and heightened levels of reality shock among newly graduated nurses. Confronting the demands of night shifts, newly graduated nurses face compounded challenges stemming from limited clinical experience, including potential deficits in theoretical knowledge, procedural skills, and acute patient assessment capabilities [38]. Physiologically, night work disrupts circadian rhythms and frequently leads to significant sleep deprivation, while psychologically, the sustained high cognitive load and responsibility for managing clinical emergencies independently contribute to substantial mental exhaustion [39]. Critically, insufficient access to timely and effective support from experienced colleagues during critical situations exacerbates feelings of inadequacy and helplessness, potentially increasing the risk of adverse events [40]. Subsequent negative performance feedback can further intensify their reality shock experience.

These findings underscore the critical need for robust support systems specifically designed to mitigate reality shock in this vulnerable cohort. Building upon successful interventions like the six-dimensional apprenticeship model implemented by Hansen et al. [41], which demonstrably reduced reality shock, we propose the establishment of structured mentorship programs focused on clinical guidance and professional skill development for newly graduated nurses. Consequently, we recommend nursing management implement multifaceted strategies: strategically limiting night shift exposure for newly graduated nurses; prioritizing their career development pathways through individualized transition plans; establishing rigorous night shift competency assessment protocols based on demonstrated work adaptability; and ensuring adequate compensatory rest periods following night shifts. Collectively, these targeted interventions are essential for alleviating reality shock and fostering successful professional integration.

#### 5.2.4 Impostor phenomenon.

The findings of this study revealed a significant positive correlation between reality shock and impostor phenomenon among newly graduated nurses, suggesting that higher levels of impostor phenomenon are associated with heightened experiences of reality shock. Impostor phenomenon, characterized by an individual’s persistent attribution of successes to external factors (e.g., luck, timing, or assistance) rather than to their own competence or effort [15], appears to critically shape this relationship. Newly graduated nurses exhibiting stronger impostor phenomenon tendencies may perceive a greater discrepancy between their acquired theoretical knowledge/professional skills and the actual demands of clinical practice. Crucially, this self-doubt, inherent in IP, may impede their ability to effectively assimilate new clinical knowledge, skills, and experiences. Consequently, they may also perceive diminished prospects for career advancement, collectively contributing to a more intense reality shock experience.

These observations align with Tseng et al. [42], who demonstrated that structured interventions (e.g., rotational learning plans, targeted training, career guidance, peer support) significantly improved outcomes like general nursing ability, work self-efficacy, and reduced occupational stress and reality shock levels in newly graduated nurses compared to controls over 12 months. Their findings further suggest that such interventions can enhance work efficiency and potentially reduce adverse events. Therefore, nursing management should proactively assess impostor phenomenon levels among newly graduated nurses. Providing targeted guidance to address specific challenges stemming from impostor phenomenon, coupled with concrete opportunities for skill development and career progression, is crucial. This multifaceted approach, as supported by evidence [42], can foster faster adaptation to the clinical environment, mitigate the severity of reality shock, and ultimately enhance professional identity and retention [43].

### 5.3 Limitations

This study has several limitations. First, the use of convenience sampling, constrained to three hospitals in one province, inherently limits the representativeness of the sample and introduces potential selection bias. As a result, the generalizability (external validity) of the findings to all newly graduated nurses across different regions or healthcare settings in China may be limited. The factors identified represent associations specific to this particular sample and context. Future research should employ random sampling strategies across multiple centers and regions with larger sample sizes to enhance external validity and confirm the generalizability of these associations. Second, the cross-sectional design, while suitable for identifying associations, precludes the establishment of causal relationships among the studied variables. For instance, the observed correlation between the impostor phenomenon and reality shock cannot determine temporality or the direction of influence. Longitudinal studies are warranted to better elucidate the temporal dynamics and potential causal mechanisms underlying these relationships. Third, and most critically, the very small sample sizes in certain demographic subgroups (most notably, nurses with technical secondary education, *n* = 2) severely compromise the reliability and generalizability of the findings for these specific groups. Consequently, any interpretations pertaining to these subgroups must be considered highly preliminary and viewed with extreme caution. Future studies should employ stratified sampling or targeted recruitment strategies to ensure adequate representation across all relevant demographic strata, which would allow for more robust and reliable subgroup analyses.

### 5.4 Implications for global nursing shortage contexts

While conducted within the specific context of China, characterized by documented nursing shortages and unique societal perceptions of the profession [26,27], this study provides new insights that extend the existing literature on newly graduated nurses’ reality shock. Specifically, our findings make a distinct contribution by empirically linking the impostor phenomenon—a widely recognized but under-investigated psychological experience among high achievers—directly to the reality shock encountered by newly graduated nurses. The identification of this interplay, alongside well-documented factors such as educational preparation mismatches and demanding work schedules (particularly night shifts), offers a more nuanced understanding of the early-career adversities that may drive turnover. Given that these psychological and professional challenges likely transcend national boundaries, our research suggests that healthcare systems globally grappling with nurse shortages could benefit from developing integrated support interventions. Such interventions should not only include robust mentorship and realistic role orientation but also explicitly incorporate strategies to identify and combat the impostor phenomenon [41,42], thereby fostering resilience and improving retention during this critical transition period.

## 6. Conclusion

This study revealed a moderately high level of reality shock among newly graduated nurses. Significant determinants associated with this phenomenon encompassed educational level, monthly income, number of night shifts per month, and impostor phenomenon. To mitigate reality shock and enhance successful career adaptation in this cohort, nursing management is advised to prioritize the development and implementation of tailored interventions. These interventions should specifically target the identified modifiable factors, with particular emphasis on addressing impostor phenomenon through strategies such as cognitive reframing, structured feedback mechanisms, and peer support programs.

## Supporting information

S1 File STROBE checklist. (DOCX)

S2 File Dataset used for the study. (XLSX)
